# Structures of *Listeria monocytogenes* MenD in ThDP-bound and *in-crystallo* captured intermediate I-bound forms

**DOI:** 10.1107/S2053230X25006181

**Published:** 2025-07-17

**Authors:** Michelle Bailey, Fiona M. Given, Ngoc Anh Thu Ho, F. Grant Pearce, Timothy M. Allison, Jodie M. Johnston

**Affiliations:** a Biomolecular Interaction Centre University of Canterbury Christchurch New Zealand; b School of Physical and Chemical Sciences University of Canterbury Christchurch New Zealand; c School of Biological Sciences University of Canterbury Christchurch New Zealand; Centre for Cellular and Molecular Biology, Hyderabad, India

**Keywords:** *Listeria monocytogenes*, menaquinone biosynthesis, SEPHCHC synthase, MenD, thiamine diphosphate-dependent enzyme, intermediate I capture

## Abstract

Menaquinones (vitamin K_2_) are a family of redox-active small lipophilic molecules that serve as vital electron carriers in many bacterial electron-transport pathways. The ThDP-dependent enzyme 2-succinyl-5-enolpyruvyl-6-hydroxy-3-cyclohexene-1-carboxylate (SEPHCHC) synthase (MenD) catalyses the first irreversible step in bacterial classical menaquinone biosynthesis via a series of reactions involving covalent ThDP-bound intermediates. We report structures of MenD from the pathogen *Listeria monocytogenes* (*Lmo*MenD) in its ThDP cofactor-bound and *in-crystallo* captured intermediate I-bound forms. Analysis of the structures revealed that *Lmo*MenD adopts the typical three-domain ThDP-dependent fold observed for MenD orthologs, while a combination of structure, size-exclusion chromatography, mass photometry and small-angle X-ray scattering analysis showed that the enzyme has a homotetrameric quaternary structure. While both of the ligand-bound structures reported here were very similar, comparison with an apo form from the PDB revealed a closing down of the active site in the ligand-bound forms, with more complete models suggesting lower levels of disorder around key regions of the active site that interface with ThDP or the captured intermediate. Enzyme kinetics characterization showed the enzyme was active and enabled allosteric inhibition to be measured. There was weak inhibition of enzyme activity in the presence of 1,4-dihydroxy-2-naphthoic acid, an allosteric regulator of *Mycobacterium tuber­culosis* MenD and downstream metabolite in the menaquinone-biosynthesis pathway.

## Abstract 2

Here, we report structures of the ThDP-dependent enzyme MenD (SEPHCHC synthase) from *L. monocytogenes* in its ThDP cofactor-bound and intermediate I-bound forms. Comparisons with an apo form previously deposited in the PDB revealed that this MenD adopts a typical three-domain ThDP-dependent fold, with lower levels of disorder associated with closing of the active site in the ligand-bound structures.

## 1. Introduction

Menaquinones, a family of related redox-active molecules, play vital functions in electron transport and energy generation in mycobacteria, Gram-positive and some anaerobically respiring Gram-negative bacteria (Nowicka & Kruk, 2010 ▸; Boersch *et al.*, 2018 ▸; Kurosu & Begari, 2010 ▸). These molecules have been implicated in other roles, such as in redox-state monitoring, influencing membrane fluidity, biofilm formation and virulence (Johnston & Bulloch, 2020 ▸; Flegler *et al.*, 2021 ▸; Mashruwala *et al.*, 2017 ▸; Honaker *et al.*, 2010 ▸; Upadhyay *et al.*, 2015 ▸). These roles, combined with the absence of menaquinone-biosynthesis pathways in humans, have made the bacterial menaquinone-biosynthesis enzymes of interest as potential drug targets (Boersch *et al.*, 2018 ▸).

**Figure 1:**
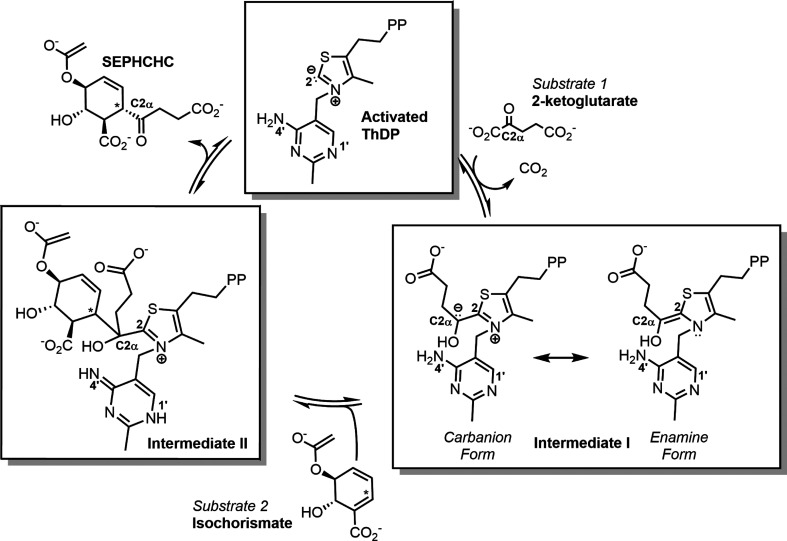
MenD catalytic cycle illustrating the formation of several ThDP-bound covalent intermediates from sequential reaction with the substrates 2-oxo­glutarate (intermediate I) and isochorismate (intermediate II), resulting ultimately in regeneration of the ThDP and release of the product SEPHCHC. Note that the aminopyrimidine ring is shown in its AP (4′-aminopyrimidine) tautomer form in ThDP and intermediate I and its IP (1′,4′-imino­pyrimidine) tautomer form in intermediate II; shuttling of tautomer states is important during the catalytic cycle.

An important player in menaquinone production is the thiamine diphosphate (ThDP)-dependent decarboxylase MenD [2-succinyl-5-enolpyruvyl-6-hydroxy-3-cyclohexene-1-carboxylate (SEPHCHC) synthase] (Johnston & Bulloch, 2020 ▸). MenD catalyses the first irreversible step of the classical menaquinone-biosynthesis pathway (Supplementary Fig. S1) via two covalent ThDP intermediates; decarboxylation of 2-oxoglutarate produces intermediate I, with subsequent addition of isochorismate generating intermediate II and breakdown to release SEPHCHC (Fig. 1 ▸; Jirgis *et al.*, 2016 ▸; Frank *et al.*, 2007 ▸; Dawson *et al.*, 2008 ▸). Like other members of the ThDP-dependent decarboxylase superfamily, MenD enzymes have a common fold consisting of the catalytic aminopyrimidine-binding (PYR) and pyrophosphate-binding (PP) domains, separated by the more divergent transhydro­genase III (TH3) domain (Vogel & Pleiss, 2014 ▸; Duggleby, 2006 ▸). All MenD structures reported to date have a tetrameric ‘dimer-of-dimers’ arrangement, and oligomerization appears to be catalytically important, with active-site formation involving the PYR and PP domains from two different monomers in each dimer (Dawson *et al.*, 2008 ▸, 2010 ▸; Jirgis *et al.*, 2016 ▸; Vogel & Pleiss, 2014 ▸; Duggleby, 2006 ▸).

The function of the TH3 domain in MenD is poorly understood. In *Mycobacterium tuberculosis* MenD (*Mtb*MenD), 1,4-dihydroxy-2-naphthoic acid (DHNA), the last cytosolic metabolite of the pathway, can bind to the TH3 domain and inhibit activity (Bashiri *et al.*, 2020 ▸). Further, DHNA inhibition, albeit less potent and without crystallographic capturing, has been reported for *Staphylococcus aureus* and *Bacillus subtilis* MenD (*Sau*MenD and *Bsu*MenD; Stanborough *et al.*, 2023 ▸; Huang *et al.*, 2024 ▸). However, due to low sequence conservation, particularly for the putative allosteric sites, it remains unclear how widespread feedback inhibition is amongst homologs (Stanborough *et al.*, 2023 ▸; Bashiri *et al.*, 2020 ▸).

Here, we report two structures of MenD from the Gram-positive pathogen *Listeria monocytogenes* (*Lmo*MenD); one with ThDP bound and a second with intermediate I bound, as well as their oligomeric states in solution and functional characterization. An existing apo *Lmo*MenD structure in the PDB (PDB entry 3lq1; New York Structural Genomics Research Consortium, unpublished work) is incomplete, showing regions of disorder around the active site and C-terminus. The liganded structures from this study are more complete, revealing that several of the previously disordered regions contribute to ligand interactions and/or closing of the active site. Interestingly, in *L. monocytogenes*, DHNA is known to fulfil respiration-independent functions in virulence and pathogenic survival (Chen *et al.*, 2019 ▸; Smith *et al.*, 2021 ▸), but the effect of DHNA on *Lmo*MenD enzyme activity had not been explored. This study has enabled us to demonstrate that while DHNA has some ability to inhibit the activity of *Lmo*MenD, this inhibition is modest.

## 2. Materials and methods

### 2.1. Macromolecule production

The *L. monocytogenes* strain 10403s *menD* gene was cloned from genomic DNA (BEI Resources) into pET-30a using NcoI and HindIII restriction sites (Table 1 ▸). The protein was overexpressed in *Escherichia coli* BL21 (DE3) cells (37°C for 2.5 h, followed by 18 h at 18°C) using Terrific Broth autoinduction medium (Stanborough *et al.*, 2023 ▸). The cells were lysed in buffer *A* [50 m*M* HEPES pH 8.0, 150 m*M* NaCl, 20 m*M* imidazole, 5%(*v*/*v*) glycerol, 0.5 m*M* TCEP] with a cOmplete Mini EDTA-free protease-inhibitor cocktail tablet using a Microfluidics M110P Microfluidiser (137 MPa). Clarified (20 000*g*, 30 min, 4°C), filtered (0.2 µm) MenD was purified using a 5 ml HP HisTrap column (Cytiva), eluting in a gradient of buffer *B* (buffer *A* with 500 m*M* imidazole). After rTEV cleavage (weight ratio 1:36) with dialysis into buffer *C* (buffer *A* without imidazole), the untagged protein was further purified by reverse IMAC and then by size-exclusion chromatography (SEC) on a HiPrep 16/60 Sephacryl S-300 HR column equilibrated with buffer *C*. Denaturing mass spectrometry measured the mass of the purified untagged protein to be 64.85 kDa, exactly matching that expected from the sequence, and *Lmo*MenD was concentrated and stored at −80°C until further use.

### 2.2. Crystallization

A MORPHEUS crystal screen dispensed by a Mosquito robot was used to find initial crystallization conditions (8.2 mg ml^−1^ MenD with 5 m*M* MgCl_2_, 1 m*M* ThDP and 1 m*M* TCEP added). Final crystals were obtained via sitting-drop vapour-diffusion fine screens uing the conditions summarized in Table 2 ▸. Intermediate I complex (PDB entry 9mnn) crystals were soaked for a minute in their crystallization condition containing 1 m*M* 2-oxoglutarate prior to cooling (Jirgis *et al.*, 2016 ▸). Further soaking of 2-oxoglutarate-soaked crystals with isochorismate, and various soaks and co-crystallizations with DHNA, were undertaken, but none of the resulting structures captured intermediate II or DHNA complexes.

### 2.3. Data collection and processing

Crystals were harvested and flash-cooled in liquid nitrogen prior to diffraction data sets being collected at the Australian Synchrotron using the MX2 macromolecular crystallography beamline equipped with a Dectris EIGER 16M detector (Aragão *et al.*, 2018 ▸). The beam was attenuated to 0–50% and 720° of data were collected. The data were processed using *X-ray Detector Software* (*XDS*; Kabsch, 2010*a* ▸,*b* ▸) via the Australian synchrotron autoprocessing pipeline. Reflections were then imported into the *CCP*4 suite (Agirre *et al.*, 2023 ▸) and merged using *AIMLESS* (Evans & Murshudov, 2013 ▸). Space-group analysis using *POINTLESS* (Evans, 2011 ▸) as part of *AIMLESS* suggested that the data were most consistent with the highest symmetry space group *P*6_2_22 or enantiomorph*P*6_4_22, with subsequent successful phasing by molecular replacement being obtained in *P*6_4_22. The data-collection and processing statistics are summarized in Table 3 ▸.

### 2.4. Structure solution and refinement

Molecular replacement was performed using *Phaser* (McCoy *et al.*, 2007 ▸), and *MATTHEWS_COEF* (Matthews, 1968 ▸; Kantardjieff & Rupp, 2003 ▸) in *CCP*4 (Agirre *et al.*, 2023 ▸) determined one molecule to be present in the asymmetric unit. The incomplete apo *Lmo*MenD structure (PDB entry 3lq1) was used as a molecular-replacement model to solve an initial, lower resolution ThDP-bound *Lmo*MenD structure, which served as the search model for the higher resolution structures that we report here (PDB entries 9e9b and 9mnn). These structures underwent iterative rounds of manual building in *Coot* (Emsley & Cowtan, 2004 ▸; Emsley *et al.*, 2010 ▸) interspersed with rounds of refinement using *REFMAC*5 (Murshudov *et al.*, 2011 ▸) in *CCP*4 followed by Cartesian annealing and further refinement using *Phenix* (Afonine *et al.*, 2012 ▸; Liebschner *et al.*, 2019 ▸). The final refinement data for both structures are presented in Table 4 ▸; while the *R*_free_ for PDB entry 9e9b is good for the resolution, that for PDB entry 9mnn is a little higher due to the incorporation of weaker higher resolution data for this structure (see the comparative CC_1/2_ values for outer shells in Table 3 ▸). However, the value of incorporating these weaker data in building the model of PDB entry 9mnn was a significant advantage and the model exhibits high-quality validation statistics, shown in Table 4 ▸, in the 99th percentiles. Structures were analysed for oligomeric assemblies (*PDBePISA*; Krissinel & Henrick, 2007 ▸) and compared with other structurally characterized MenD enzymes (*PBDeFold*; Krissinel & Henrick, 2004 ▸).

### 2.5. SAXS

Small-angle X-ray scattering (SAXS) data were collected at the Australian Synchrotron on the SAXS/WAXS beamline (Kirby *et al.*, 2013 ▸; Ryan *et al.*, 2018 ▸). Experimental and processing details and the resulting data are provided in the supporting information.

### 2.6. Mass photometry

Measurements were performed on a TwoMP mass photometer (Refeyn; Wu & Piszczek, 2021 ▸), with further details and data provided in the supporting information.

### 2.7. Differential scanning fluorimetry (DSF)

The thermal stability of *Lmo*MenD in its apo and ThDP-bound (300 m*M* ThDP) forms was determined using a QuantStudio3 Real-Time polymerase chain-reaction instrument, with further details and data provided in the supporting information.

### 2.8. UV/Vis activity and DHNA inhibition assays

The UV/Vis-based kinetic assays measuring the consumption of the second MenD substrate, isochorismate (ɛ_278_ = 8300 *M*^−1^ cm^−1^), were adapted from previously established methods (Bashiri *et al.*, 2020 ▸; Ho *et al.*, 2025 ▸). Using this method, the *K*_m_ values for ThDP, 2-oxoglutarate and isochorismate and *k*_cat_ were determined, and the inhibitory effect of DHNA was measured. Complete methods and data are provided in the supporting information.

## 3. Results and discussion

### 3.1. *Lmo*MenD is an active SEPHCHC synthase that can competently bind ligands

**Figure 2:**
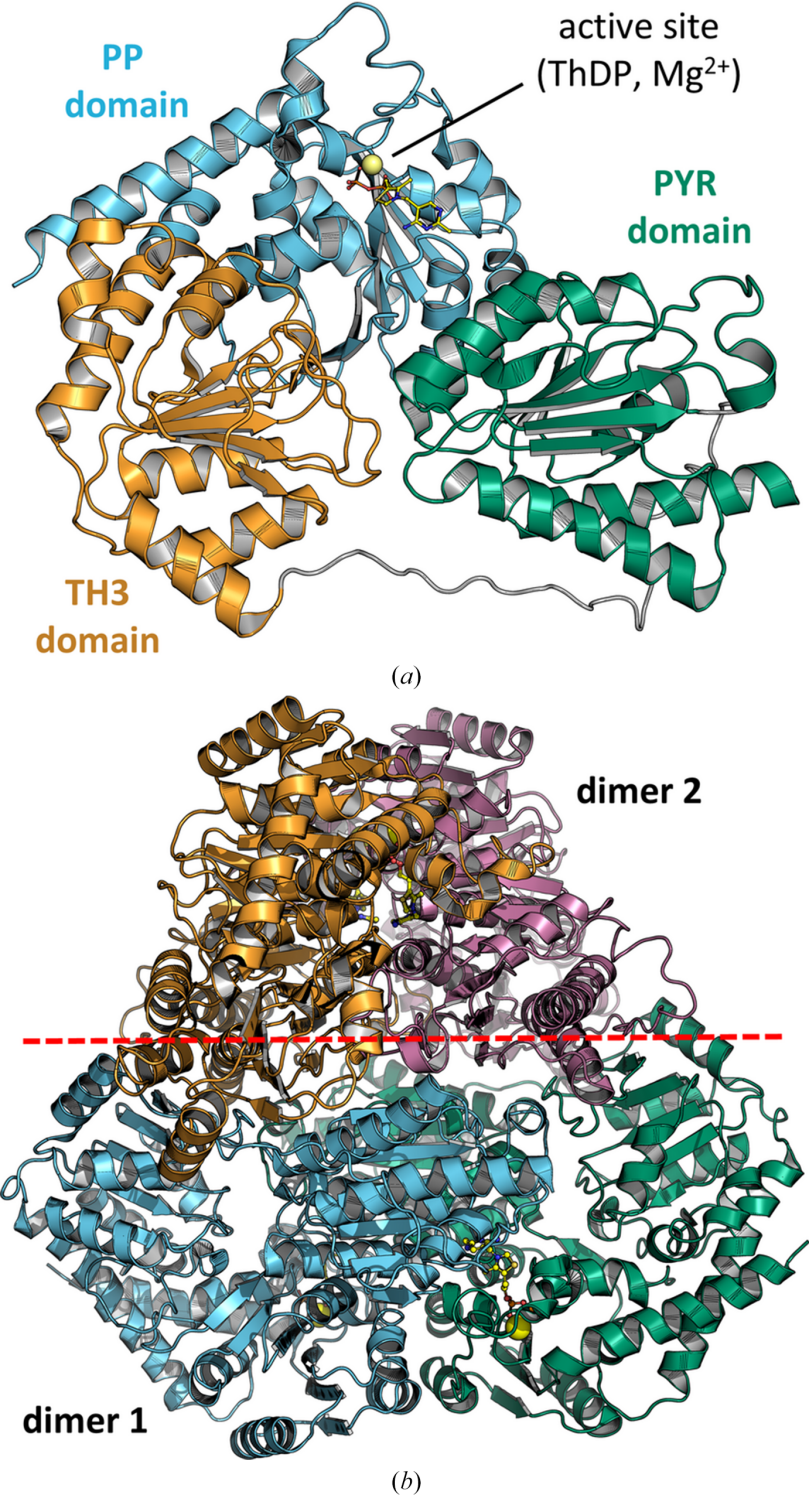
(*a*) *Lmo*MenD monomer (PDB entry 9e9b) with PYR (green), TH3 (orange) and PP (blue) domains. (*b*) Dimer-of-dimers *Lmo*MenD tetramer (PDB entry 9e9b; dimer 1, blue and green; dimer 2, orange and rose).

**Figure 3:**
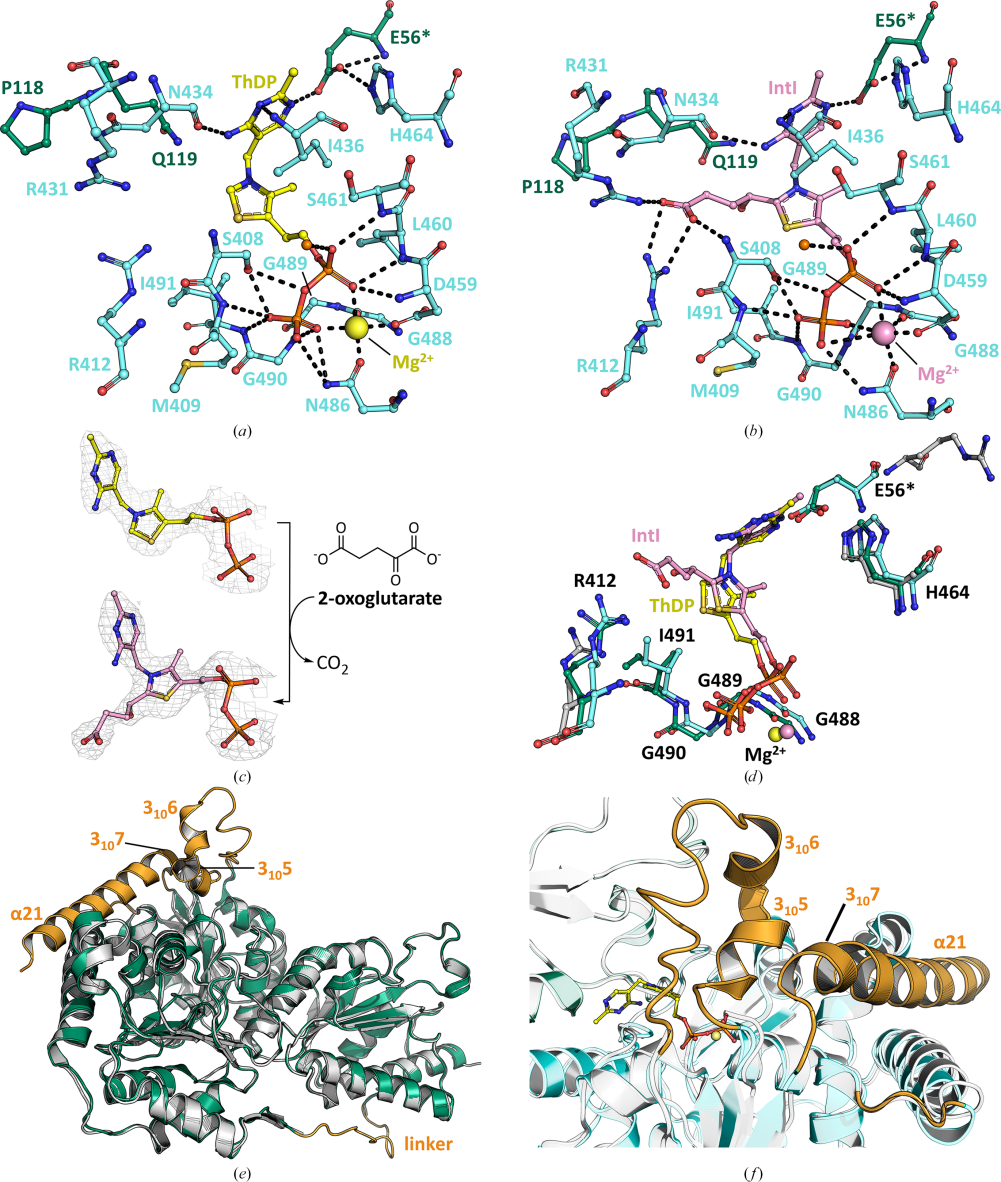
(*a*) ThDP and Mg^2+^ (yellow) binding in the *Lmo*MenD (PDB entry 9e9b) active site. (*b*) Intermediate I (IntI; rose) formation in the *Lmo*MenD (PDB entry 9mnn) active site. The residues comprising the active site originate from the PYR (green) and PP (blue) domains, with the catalytic glutamate (Glu56) highlighted (*). Water molecules are depicted as orange spheres and polar contacts as black dashes. (*c*) ThDP (PDB entry 9e9b; yellow) and intermediate I (PDB entry 9mnn; rose) in their respective 2*mF*_o_ − *DF*_c_ electron-density maps (contoured at 1σ; grey). (*d*) Overlay of the apo (PDB entry 3lq1; grey), ThDP-bound (PDB entry 9e9b; green) and intermediate I-bound (PDB entry 9mnn; blue) active sites, with residues differing between the structures shown as sticks. (*e*) Overlay of the apo (PDB entry 3lq1; grey) and ThDP-bound (PDB entry 9e9b; green) *Lmo*MenD monomers, with regions becoming ordered upon cofactor binding highlighted in orange. (*f*) Overlay of the apo (PDB entry 3lq1; grey) and ThDP-bound (PDB entry 9e9b; PYR domain, green; PP domain, blue) active sites, with regions becoming ordered upon cofactor binding highlighted in orange and ThDP in the active site shown as yellow sticks.

To understand how cofactor binding may affect the structure of *Lmo*MenD, we overexpressed and then purified recombinant N-terminally His-tagged *Lmo*MenD by affinity chromatography. Following tag removal, and further purification by reverse affinity chromatography and SEC, the SEPHCHC enzyme activity of *Lmo*MenD was kinetically characterized with respect to the ThDP cofactor and the two substrates 2-oxoglutarate and isochorismate (Supplementary Table S1 and Fig. S2). Having produced active enzyme, we pursued a ThDP-bound *Lmo*MenD structure (PDB entry 9e9b; Figs. 2 ▸, 3 ▸*a* and 3 ▸*c*–3 ▸*f*, Table 4 ▸), followed by attempts to capture *in-crystallo* intermediate I formation. Adapting an approach that had been successful for *Mtb*MenD (Jirgis *et al.*, 2016 ▸), where 2-oxoglutarate was soaked into the co-crystals immediately prior to cryocooling, the intermediate I-bound *Lmo*MenD structure (PDB entry 9mnn; Figs. 3 ▸*b*–3 ▸*d*, Table 4 ▸) was able to be captured. These structures allowed us to characterize the binding interactions of the ThDP and intermediate I ligands, as well as the associated conformational changes in comparison to the apo structure in the PDB (PDB entry 3lq1; see Supplementary Table S3 for comparison of crystal conditions *etc.* with our structures).

### 3.2. The *Lmo*MenD monomer adopts a typical three-domain ThDP-dependent enzyme fold which has greater order in the ligand-bound structures

ThDP-bound and intermediate I-bound *Lmo*MenD, like apo *Lmo*MenD, possess a typical ThDP-dependent enzyme fold comprised of a PYR domain (residues 1–174), a TH3 domain (residues 208–371) and a PP domain (residues 372–580) (Fig. 2 ▸*a*; Dawson *et al.*, 2008 ▸, 2010 ▸; Jirgis *et al.*, 2016 ▸; Stanborough *et al.*, 2023 ▸). Each domain contains six parallel β-strands sandwiched between six (PYR, TH3) or eight (PP) α-helices (Supplementary Figs. S3 and S4). The PYR and TH3 domains are connected by a flexible linker (residues 175–207), whilst the TH3 and PP domains are connected by the α13 helix (Supplementary Figs. S3 and S4). Structural comparison with other MenD structures affirmed significant structural conservation (r.m.s.d. of 1.2–2.0 Å across all C^α^ atoms; Supplementary Table S2); however, some differences across the structures are apparent. In particular, *Lmo*MenD differs in its linkage between the PYR and TH3 domains [flexible linker and following (α7)], the flexible PP domain region (residues 487–512) closing on the active site, and an elongated final C-terminal helix (α21) which, as in *Bsu*MenD and *Sau*MenD, is longer than the equivalent in *Mtb*MenD and *E. coli* MenD (*Eco*MenD).

Comparison between the apo *Lmo*MenD (PDB entry 3lq1) and our ThDP-bound and intermediate I-bound structures (r.m.s.d. of 0.34 Å for ThDP and intermediate I, r.m.s.d. of 0.5–0.6 Å for apo and ThDP/intermediate I across all C^α^ atoms; Krissinel & Henrick, 2004 ▸) showed a similar fold, but reinforced that both of the ligand-bound structures were more complete; all of the disordered regions missing from the apo structure (Supplementary Table S3) were able to be modelled in the ThDP-bound and intermediate I-bound structures (Supplementary Table S3 and Fig. S5). These regions include the linker (chain *A*, 189–200; chain *B*, 190–200), a flexible region following the α13 helix (chain *A*, 381–383), the flexible PP domain region that closes on the active site (487–512, including 3_10_-helices 5–7), as well as the C-terminus (556–580, helix α21) (Fig. 3 ▸*e*). In ligand-bound structures, the flexible 487–512 region was in a closed and ordered form, making interactions with the ligands (Fig. 3 ▸*f*). Additionally, parts of the C-terminus (residues 556–565) missing in the apo structure made interactions with the 487–512 region, thus supporting the closing of the active site in the ligand-bound structures (Fig. 3 ▸*f*). Whilst there are differences between the apo and ligand-bound structures, such as the source protein, crystal conditions and space group (summarized in Supplementary Table S3 and Fig. S6), which we cannot rule out as a source of differences, there are precedents for increased order in ligand-bound MenD structures. Comparable levels of disorder affecting parts of the active site were observed for apo *Mtb*MenD (PDB entry 5ery) and *Eco*MenD (PDB entry 3flm), with binding of ThDP associated with conformational changes assisting in active-site formation and an overall decrease in disorder (Priyadarshi, Kim *et al.*, 2009 ▸; Priyadarshi, Saleem *et al.*, 2009 ▸; Jirgis *et al.*, 2016 ▸).

### 3.3. *Lmo*MenD is a symmetrical tetramer (dimer of dimers) in the crystal form

The apo *Lmo*MenD structure (PDB entry 3lq1, space group *P*4_3_2_1_2) contains two monomers, one from each dimer, in the asymmetric unit. Upon the application of crystallographic symmetry, a tetramer composed of a dimer of dimers is formed. Similarly, the ThDP-bound and intermediate I-bound structures (space group *P*6_4_22) form the same dimer-of-dimers arrangement upon the application of crystallographic symmetry to the monomer present in the asymmetric unit (Fig. 2 ▸*b*). *PISA* analysis (Krissinel & Henrick, 2007 ▸) suggests that the dimeric unit buries ∼23% of its surface area, primarily from interactions between the PYR and PP domains from different monomers. The tetramer buries a total of ∼42% of the available surface area through additional interactions between the TH3 domains and a protruding loop region (108–119) from the PYR domain. The *Lmo*MenD structures are consistent with the tetrameric dimer-of-dimer arrangements observed in other MenD structures (Stanborough *et al.*, 2023 ▸; Jirgis *et al.*, 2016 ▸; Dawson *et al.*, 2008 ▸, 2010 ▸) and the tetrameric arrangement was detected in solution by SEC and mass photometry (Supplementary Fig. S7). Additionally, mass photometry analysis and DSF indicate no changes in oligomerization (Supplementary Fig. S7) or thermostability (Supplementary Fig. S8; the *T*_m_ values were 56.0 ± 0.2°C for the apo form, 56.1 ± 0.1°C for Mg^2+^ and 56.4 ± 0.1°C for ThDP), respectively, in the presence of ThDP or under the conditions in which intermediate I may form, suggesting that the binding of these ligands causes no significant changes in oligomerization. Consistent with these observations, SAXS analysis showed that the experimental scattering curve for the apo protein was well fitted by the tetrameric ThDP-bound crystal structure (PDB entry 9e9b), with a χ^2^ value of 0.27, supporting the predominance of a tetrameric assembly in solution (Supplementary Fig. S9).

### 3.4. ThDP and intermediate I binding to *Lmo*MenD

In ThDP-dependent enzymes such as MenD, ThDP plays a vital role in catalysis due to both its substrate-binding and chemical features; it adopts a V-shaped conformation upon enzyme binding which brings the N4′ group of its aminopyrimidine ring into the proximity of the C2 atom of its thiazolium ring, enabling proton abstraction and formation of the activated ThDP ylide (Frank *et al.*, 2007 ▸). In most ThDP-dependent enzymes there is a conserved glutamic acid which facilitates tautomerization of the aminopyrimidine ring, enabling the shuttling of a proton during catalytic cycling (Balakrishnan *et al.*, 2012 ▸). Analysis of the ThDP-bound *Lmo*MenD structure suggests that the active-site configuration and ThDP-binding interactions are similar to those observed in other MenD enzymes. The ThDP diphosphate group tethers the cofactor to the enzyme via interactions with a divalent magnesium cation and the PP domain; specifically, residues Ser408, Met409, Asp459, Leu460 and Ser461, as well as Asn486 and Ile491, from the region that closes on the active site (Fig. 3 ▸*a*). The ThDP aminopyrimidine ring sits between the PP and PYR domains, making hydrogen-bonding interactions with Glu56, Gln119, Asn434 and Ile436 (Fig. 3 ▸*a*). The conserved residue Glu56 is likely to be involved in tautomerization of the aminopyrimidine ring during catalysis, and Ile436 is likely to be responsible for the bent ThDP conformation important for ThDP activation and catalysis, while Gln119 has speculative roles in many steps, including product release (Dawson *et al.*, 2008 ▸, 2010 ▸; Jirgis *et al.*, 2016 ▸; Stanborough *et al.*, 2023 ▸; Priyadarshi, Kim *et al.*, 2009 ▸; Priyadarshi, Saleem *et al.*, 2009 ▸).

The reaction cycle for ThDP-dependent decarboxylase enzymes such as MenD is through a series of ThDP-linked intermediates; a transient pre-decarboxylation intermediate forms after the C2 atom of the thiazolium ring of the ThDP ylide reacts with of the first (α-ketoacid) substrate (for MenD this is 2-oxoglutarate). The metastable intermediate I subsequently forms after decarboxylation, and is traditionally considered to be in resonance between an enamine and a carbanion (centred on C2α) form (Fig. 1 ▸). It has been observed for *Eco*MenD and *Mtb*MenD that intermediate I appears to preferentially adopt the tetrahedral form (Jirgis *et al.*, 2016 ▸; Song *et al.*, 2016 ▸). For *Ec*oMenD this strained tetrahedral form may be reversibly protonated by the aminoprymidine N4′ proton, which is near C2α (C2α to N4′ distance of 3.0 Å in intermediate I-captured *Eco*MenD; Song *et al.*, 2016 ▸). Intermediate I then goes on to react via C2α with the second substrate isochorismate to form intermediate II (Fig. 1 ▸). Proton transfer from the aminopyrimidine ring of the ThDP assists with the subsequent breakdown of intermediate II to release product. In *Mtb*MenD, where this second intermediate was also captured, there is a noted movement of C2α-OH towards the aminopyrimidine N4′ (from 4.5 Å in intermediate I to 2.3 Å in intermediate II), which is proposed to facilitate product release only when the appropriate second intermediate has formed (Jirgis *et al.*, 2016 ▸). The intermediate I-bound *Lmo*MenD structure has clearly defined intermediate I electron density (Fig. 3 ▸*c*), supporting a tetrahedral conformation at C2α, similar to that observed for intermediate I-bound *Mtb*MenD and *Eco*MenD, and supporting the general idea that MenD enzymes favour the carbanion or transiently protonated form of intermediate I (Jirgis *et al.*, 2016 ▸; Qin *et al.*, 2018 ▸; Song *et al.*, 2016 ▸). To further support the similarity of the intermediate I conformation between species, in *Lmo*MenD C2α is 3.21 Å from N4′, a similar distance to that observed in intermediate I-bound *Eco*MenD. C2α-OH is 4.41 Å from the aminopyrimidine N4′, similar to the distance observed in intermediate I-bound *Mtb*MenD, suggesting that in *Lmo*MenD, like the other MenDs, the captured form of intermediate I is primed for addition of the next substrate, rather than in a position to support premature product release.

Analysis of intermediate I binding reveals that the shared parts of ThDP and intermediate I bind very similarly to the active site (Figs. 3 ▸*a*, 3 ▸*b* and 3 ▸*d*), with the intermediate I-specific 2-oxoglutarate-derived terminal carboxylate group making additional interactions with the active-site residues Ser408, Arg412 and Arg431 (Fig. 3 ▸*b*). Equivalents to Arg412 and Arg431 in other MenDs were identified to play a crucial role in orienting intermediate I for catalysis (Qin *et al.*, 2018 ▸; Dawson *et al.*, 2010 ▸). In *Lmo*MenD, the side chain of Asn434 sits 3.6 Å from the intermediate I terminal carboxylate, mirroring the configuration of the *Bsu*MenD and *Sau*MenD active sites. In *Eco*MenD and *Mtb*MenD, a spatially equivalent intermediate I-interacting asparagine originates from the PYR domain, next to the conserved glutamine, with *Bsu*MenD, *Sau*MenD and *Lmo*MenD containing a proline (*Lmo*MenD Pro118) at this position (Figs. 3 ▸*a* and 3 ▸*b*; Dawson *et al.*, 2010 ▸; Jirgis *et al.*, 2016 ▸). Despite the closing of the active site observed in our *Lmo*MenD ThDP and intermediate I structures, there appears to be a tunnel to the surface from the active site (with the terminal carboxylate tail of intermediate I visible; Supplementary Fig. S10). This tunnel is lined with residues inferred by homology to interact with isochorismate/the isochorismate portion of intermediate II (for example Ser34, Arg35, Arg108, Gln119 and Arg301), suggesting that even in this closed form the enzyme can support access of substrate two.

### 3.5. Allosteric site and DHNA inhibition

To explore whether *Lmo*MenD enzyme activity is sensitive to inhibition by DHNA, activity assays were performed with and without this downstream metabolite (Supplementary Fig. S2*d*). Due to the solubility challenges of DHNA and limitations of the assay, a saturated IC_50_ curve could not be obtained; however, a decrease of 34% in enzymatic activity (66% residual activity) was detected in the presence of 12.5 µ*M* DHNA (Supplementary Fig. S2*d*). While conditions vary, other enzymes with DHNA inhibition characterized to date (*i.e. Mtb*MenD and *Sau*MenD) appear to be inhibited more potently, both with measurable IC_50_ values: 53 n*M*for *Mtb*MenD and 2.3/3.7 µ*M* for *Sau*MenD (two different strains) (Stanborough *et al.*, 2023 ▸; Bashiri *et al.*, 2020 ▸). From a biological perspective it is uncertain what this magnitude of inhibition of *Lmo*MenD by DHNA will mean. For *Sau*MenD, which had a decrease of 82–87% in enzymatic activity (13–18% residual activity) in the presence of 12.5 µ*M* DHNA, it was found that 50 µ*M* DHNA impacted the growth of all four *S. aureus* strains tested and 150 µ*M* abolished bacterial growth entirely, but it could be rescued by the addition of menaquinone-4 (Stanborough *et al.*, 2023 ▸). However, each bacterium is different, and it is known that in *L. monocytogenes* DHNA has additional respiratory-independent functions that contribute to pathogen survival (Chen *et al.*, 2019 ▸; Smith *et al.*, 2021 ▸); thus, any regulatory networks involving DHNA may be more complex.

**Figure 4:**
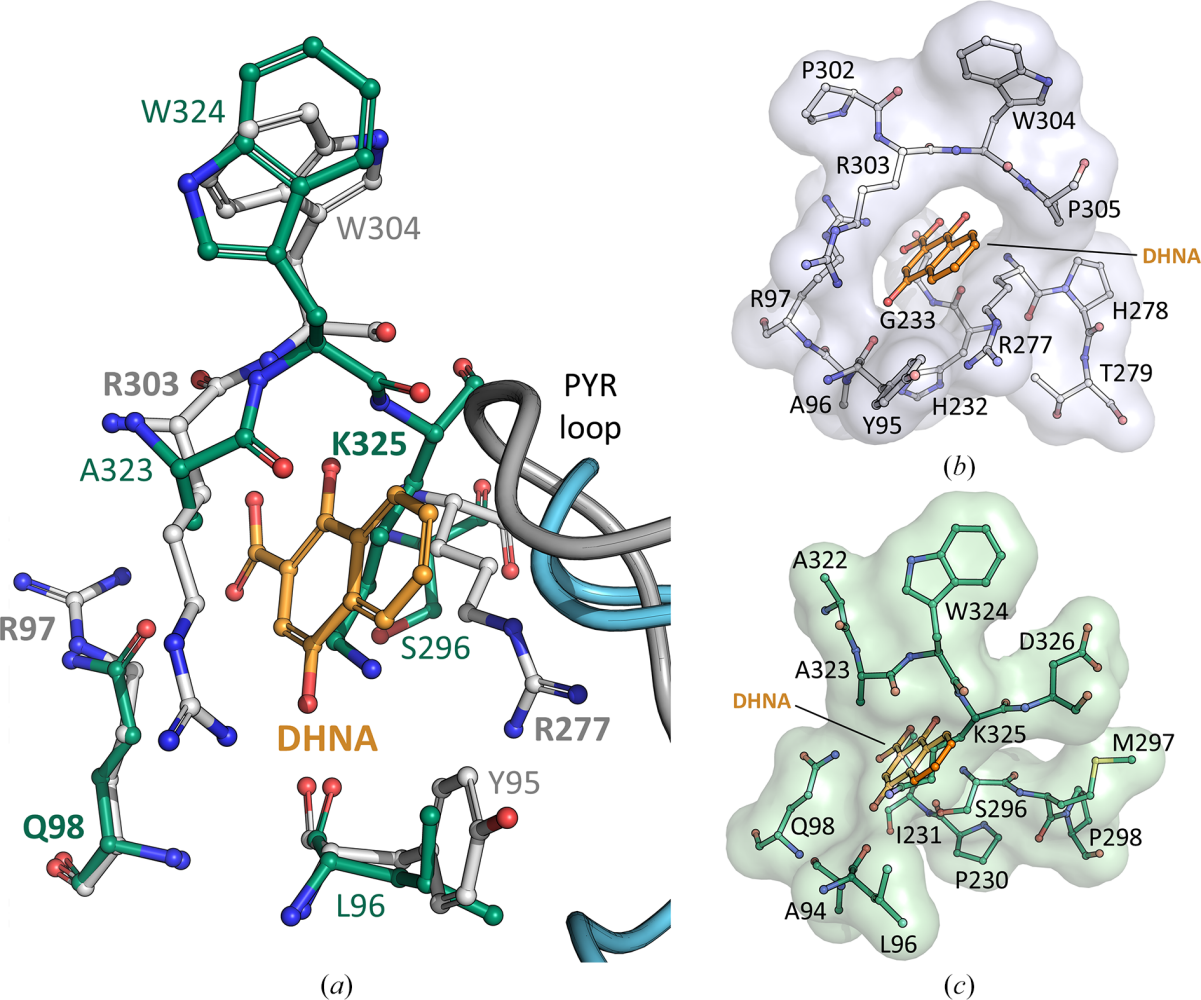
(*a*) Overlay of the putative *Lmo*MenD (PDB entry 9e9b; green) and *Mtb*MenD (PDB entry 6o0j; grey) DHNA (orange) binding sites. Residues hypothesized to fulfil equivalent functions to a particular *M. tuberculosis* arginine-cage constituent are in bold, with *Lmo*MenD Lys325 potentially able to fulfil the function of *Mtb*MenD Arg277 or Arg303. (*b*) Surface representation of the *Mtb*MenD (PDB entry 6o0j; grey) allosteric site showing the DHNA (orange) binding pocket. (*c*) Surface representation of the *Lmo*MenD (PDB entry 9mnn; green) putative allosteric site, highlighting the requirement of induced-fit conformational changes to accommodate DHNA (orange) binding.

Attempts to obtain a DHNA-bound *Lmo*MenD structure were unsuccessful, matching the difficulties experienced for *Sau*MenD (Stanborough *et al.*, 2023 ▸), leaving *Mtb*MenD as the only MenD for which DHNA binding has been captured crystallographically (Bashiri *et al.*, 2020 ▸). As for *Sau*MenD (Stanborough *et al.*, 2023 ▸), it appears that the allosteric site conformation captured in our crystal structures of *Lmo*MenD would not be directly suitable for DHNA binding. Superpositions of the ThDP-bound *Lmo*MenD (PDB entry 9e9b) and the ThDP/DHNA-bound *Mtb*MenD (PDB entry 6o0j) structures suggests that binding of DHNA to the putative allosteric site may be possible (Fig. 4 ▸), but for this to be the case there would need to be structural rearrangements of the pocket, particularly Lys325, which blocks the DHNA-binding cavity in its current conformation (Fig. 4 ▸*c*). Whilst the arginine-cage residues are important for DHNA binding in *Mtb*MenD outside mycobacteria and *Rhodococcus*, these residues are poorly conserved (Bashiri *et al.*, 2020 ▸). Site-directed mutagenesis of *Sau*MenD suggested that allosteric inhibition by DHNA was possible if other residues could fulfil similar roles (Stanborough *et al.*, 2023 ▸). Assuming that induced-fit conformational changes to accommodate DHNA binding take place, Gln98 and Lys325 may be able to act as functional equivalents to Arg97 and Arg277, respectively, of the *Mtb*MenD arginine cage (Fig. 4 ▸ and Supplementary Fig. S3). This would require marginal movement of Gln98 but larger conformational movements of Lys325 and surrounding regions including Ala323–Asp326; recent work with wild-type *Mtb*MenD and several allosteric site mutants has shown that DHNA can affect the conformation and stability of the equivalent region (Arg303–Asp306) in *Mtb*MenD (Ho *et al.*, 2025 ▸). However, future research, including mutagenesis and attempts to capture binding crystallographically, are required to test this hypothesis, and until then the significance and mechanism of action of DHNA on *Lmo*MenD enzyme activity will remain unclear.

## 4. Conclusion

Structural characterization of two ligand-bound forms (ThDP/intermediate I) of *Lmo*MenD, complemented by the determination of in-solution oligomerization behaviour, revealed the conservation of monomer fold and tetrameric quaternary structure between this MenD and others characterized to date. Comparison with a previously deposited but unpublished apo structure of *Lmo*MenD revealed changes in key regions, resulting in a more closed active site in ligand-bound structures. *Lmo*MenD SEPHCHC synthase activity was detected,with inhibition by the *Mtb*MenD allosteric regulator DHNA observed.

## 5. Related literature

The following references are cited in the supporting information for this article: Madeira *et al.* (2024 ▸), Manalastas-Cantos *et al.* (2021 ▸) and Robert & Gouet (2014 ▸).

## Supplementary Material

PDB reference: *Listeria monocytogenes* MenD with Mg^2+^ and ThDP bound, 9e9b

PDB reference: with Mg^2+^ and intermediate I bound, 9mnn

Supplementary Methods, Supplementary Figures and Tables. DOI: 10.1107/S2053230X25006181/us5161sup1.pdf

## Figures and Tables

**Table 1:** Macromolecule-production information

Source organism	*Listeria monocytogenes* (strain 10403s)
DNA source	Genomic DNA
Forward primer†	TTTCAGGGCGCCATGGTGACAAACCACGAACAAGTGCTG
Reverse primer‡	GTGCGGCCGCAAGCTTTTAATTTAATGCCTTCAACGCGTCC
Expression vector	pET-30a
Expression host	*Escherichia coli* BL21(DE3)
Complete aa sequence of the construct produced§	MHHHHHHSSGLVPRGSGMKETAAAKFERQHMDSPDLDLGTGSENLYFQ**GA**MVTNHEQVLTDYLAAFIEELVQAGVKEAIISPGSRSTPLALMMAEHPILKIYVDVDERSAGFFALGLAKASKRPVVLLCTSGTAAANYFPAVAEANLSQIPLIVLTADRPHELRNVGAPQAMDQLHLYGSHVKDFTDMALPENSEEMLRYAKWHGSRAVDIAMKTPRGPVHLNFPLREPLVPILEPSPFTATGKKHHHVHIYYTHEVLDDSSIQKMVTECTGKKGVFVVGPIDKKELEQPMVDLAKKLGWPILADPLSGLRSYGALDEVVIDQYDAFLKEAEILDKLTPEVVIRFGSMPVSKPLKNWLEQLSDIRFYVVDPGAAWKDPIKAVTDMIHCDERFLLDIMQQNMPDDAKDAAWLSRWTSYNKVAREIVLAEMANTTILEEGKIVAELRRLLPDKAGLFIGNSMPIRDVDTYFSQIDKKIKMLANRGANGIDGVVSSALGASVVFQPMFLLIGDLSFYHDMNGLLMAKKYKMNLTIVIVNNDGGGIFSFLPQANEPKYFESLFGTSTELDFRFAAAFYDADYHEAKSVDELEEAIDKASYHKGLDIIEVKTNRHENKANHQALWAKIADALKALN

† The NcoI restriction site is underlined.

‡ The HindIII restriction site is underlined.

§ The part of the His-tag that is removed after TEV cleavage is underlined. Tag residues remaining after cleavage are highlighted in bold.

**Table 2:** Crystallization

Method	Vapour diffusion
Plate type	Sitting drop
Temperature (K)	291
Protein concentration (mg ml^−1^)	8.2
Buffer composition of protein solution	50 m*M* HEPES pH 8.0, 150 m*M* NaCl, 15% glycerol, 5 m*M* MgCl_2_, 1 m*M* TCEP, 1 m*M* ThDP
Composition of reservoir solution	
PDB entry 9e9b	12.5%(*w*/*v*) PEG 1000, 12.5%(*w*/*v*) PEG 3350, 12.5% MPD, 0.02 *M* 1,6-hexanediol, 0.02 *M* butan-1-ol, 0.02 *M* (*RS*)-1,2-propanediol, 0.02 *M* propan-2-ol, 0.02 *M* butane-1,4-diol, 0.02 *M* propane-1,3-diol, 0.1 *M* MOPS/HEPES pH 7.5
PDB entry 9mnn	5%(*w*/*v*) PEG 4000, 20%(*v*/*v*) glycerol, 0.03 *M* MgCl_2_, 0.03 *M* CaCl_2_, 0.1 *M* MES/imidazole pH 6.3
Volume and ratio of drop	
PDB entry 9e9b	300 nl (1:1 ratio)
PDB entry 9mnn	2 µl (1:1 ratio)
Volume of reservoir (µl)	30

**Table 3:** Data collection and processing Values in parentheses are for the outer resolution shell.

Structure	*Lmo*MenD–ThDP (PDB entry 9e9b)	*Lmo*MenD–intermediate I (PDB entry 9mnn)
Diffraction source	MX2 beamline, Australian Synchrotron	MX2 beamline, Australian Synchrotron
Wavelength (Å)	0.953700	0.953700
Space group	*P*6_4_22	*P*6_4_22
*a*, *b*, *c* (Å)	176.03, 176.03, 100.14	175.23, 175.23, 100.23
α, β, γ (°)	90, 90, 120	90, 90, 120
Mosaicity	0.13	0.18
Resolution range	47.57–2.61 (2.73–2.61)	47.59–2.76 (2.91–2.76)
No. of unique reflections	28314 (3395)	23604 (3200)
No. of observations	1542504 (191122)	737371 (91801)
Multiplicity	54.5 (56.3)	31.2 (28.7)
*R*_merge_ (all *I*−/*I*+)	0.381 (2.935)	0.425 (3.075)
*R*_p.i.m._ (all *I*+/*I*−)	0.052 (0.393)	0.077 (0.559)
CC_1/2_	0.994 (0.760)	0.996 (0.384)
〈*I*/σ(*I*)〉	14.6 (2.1)	10.3 (1.4)†
Completeness (%)	100 (100)	99.1 (94.4)
Wilson *B* factor (Å^2^)	42.56	58.10

† The mean *I*/σ(*I*) in the outer shell falls below 2.0 at resolutions above 2.92 Å. Merged CC_1/2_ correlations between intensity estimates from half data sets (Karplus & Diederichs, 2015 ▸) were analysed and influenced the high-resolution cutoff for data processing.

**Table 4:** Structure refinement

Structure	*Lmo*MenD–ThDP (PDB entry 9e9b)	*Lmo*MenD–intermediate I (PDB entry 9mnn)
Resolution range (Å)	47.57–2.61 (2.70–2.61)	45.16–2.79 (2.91–2.79)
Completeness (%)	99.94 (100.00)	99.55 (98.00)
σ Cutoff	1.350	1.340
No. of reflections		
Working set	26868 (2630)	21854 (2616)
Test set	1410 (148)	1166 (139)
*R*_work_	0.1991 (0.2714)	0.1868 (0.2915)
*R*_free_	0.2353 (0.3240)	0.2502 (0.3946)
No. of non-H atoms		
Total	4628	4606
Protein	4535	4544
Ligand	1 TPP, 1 Mg^2+^	1 TD6, 2 Mg^2+^
Solvent	66	27
R.m.s. deviations		
Bond lengths (Å)	0.003	0.008
Angles (°)	0.528	1.021
Average *B* factors (Å^2^)		
Protein	55.86	56.85
Ligand	55.70	50.51
Solvent	49.82	66.98
Ramachandran plot		
Most favoured (%)	95.10	94.97
Allowed (%)	4.90	5.03
*MolProbity* score	1.58 [99th percentile]	2.04 [99th percentile]
*MolProbity* clashscore	4.71 [99th percentile]	8.32 [99th percentile]
