# Supplementary material for: Structures of Listeria monocytogenes MenD in ThDP-bound and in-crystallo captured intermediate I-bound forms
Source: Acta Crystallogr F Struct Biol Commun. 2025 Jul 17;81(Pt 8):348–57. doi: 10.1107/S2053230X25006181 (PMC12312563; doi:10.1107/S2053230X25006181)
Supplement: Supplementary file 1 [file f-81-00348-sup1.pdf]

Acta Cryst F

STRUCTURAL BIOLOGY
COMMUNICATIONS

Volume 81 (2025)

Supporting information for article:

The structures of *Listeria monocytogenes* MenD in ThDP-bound and *in-crystallo* captured intermediate I-bound forms

Michelle Bailey, Fiona M. Given, Ngoc Anh Thu Ho, F. Grant Pearce, Timothy M Allison and Jodie M. Johnston

Supporting Information: Introduction

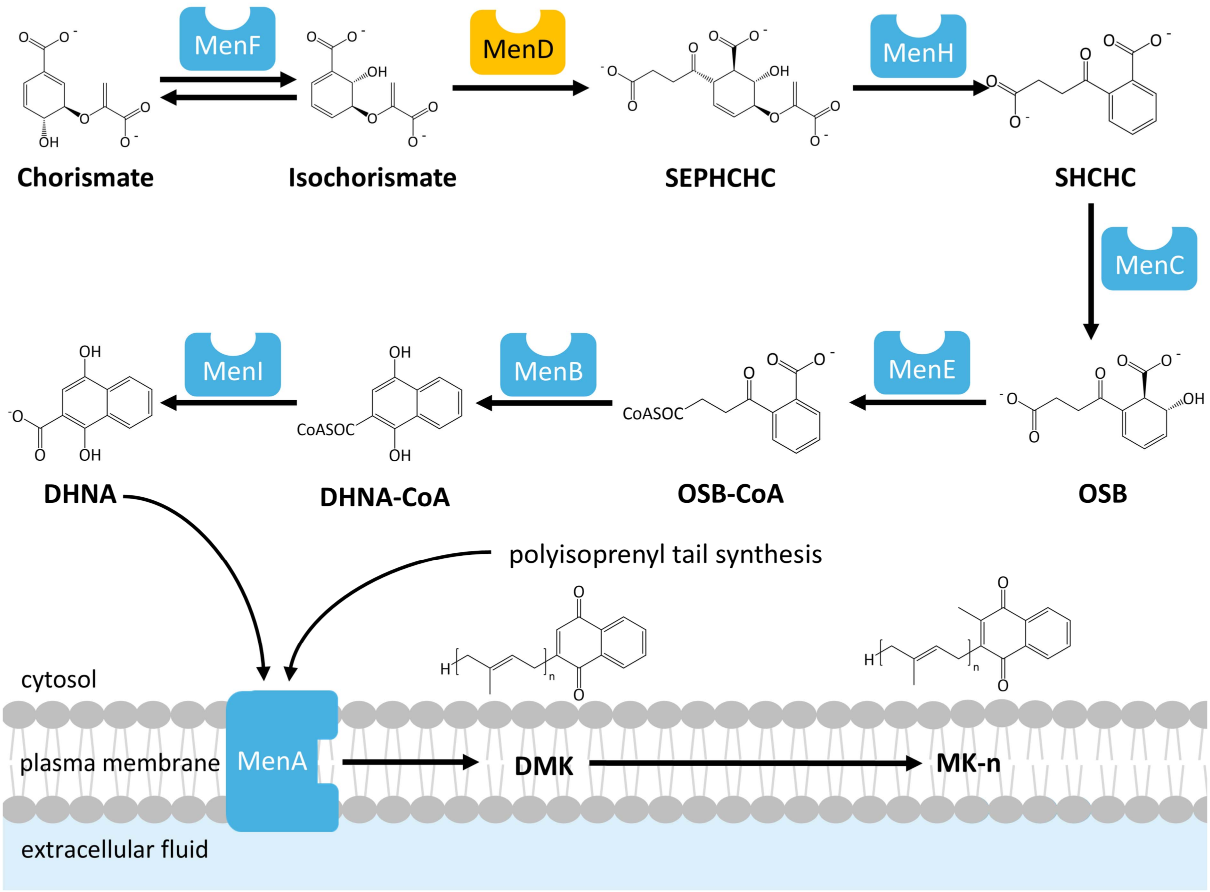

Figure S1 The classical menaquinone biosynthesis pathway comprised of nine enzyme steps with MenD (yellow) catalysing the first irreversible reaction. MenF: isochorismate synthase; MenD: 2-succinyl-5-enolpyruvyl-6-hydroxy-3-cyclohexene-1-carboxylate (SEPHCHC) synthase; MenH: 2-succinyl-6-hydroxy-2,4-cyclohexadiene-1-carboxylate (SHCHC) synthase; MenC: o-succinylbenzoate (OSB) synthase; MenE: OSB-CoA ligase; MenB: 1,4-dihydroxy-2-naphthoic acid (DHNA)-coenzyme A (CoA) synthase; MenI: DHNA-CoA hydrolase; MenA: DHNA isoprenyltransferase; MenG: 2-demethylmenaquinone (DMK) methyltransferase; MK-n: menaquinone with variable number of isoprenyl units (n) in side chain.

Supporting Information: Methods

For Section 2.5: SAXS (Small-angle X-ray scattering)

SAXS data (Figure S9) were collected at the Australian Synchrotron on the SAXS/WAXS beamline (Ryan *et al.*, 2018, Kirby *et al.*, 2013) using a Superdex 200 Increase 5/150 GL SEC column

equilibrated in buffer (50 mM HEPES pH 7.8, 150 mM NaCl, 5 mM $MgCl_2$ and 5% glycerol). *Lmo*MenD (60 µL; 9 mg/mL) was injected at a flow rate of 0.5 mL/min at 12 °C, and eluate was X-ray irradiated (1.0332 Å) once every second. The data were collected using a Pilatus 2M detector (1.6 m distance), and reduced using scatterbrain version 2.82. Data analysis was performed using the ATSAS software package version 3.1.1 (Manalastas-Cantos et al., 2021) with Chromixs (Panjkovich & Svergun, 2018) and PRIMUS (Manalastas-Cantos et al., 2021). The X-ray crystallography structures reported here were fitted to the SAXS scattering curves with CRYSOL (Franke et al., 2017) for cross-validation of the oligomeric state of the enzyme in solution.

For Section 2.6 Mass Photometry (MP)

Measurements were performed on a TwoMP mass photometer (Refeyn) (Wu & Piszczek, 2021) and *Lmo*MenD was droplet diluted in buffer (50 mM HEPES pH 7.8, 150 mM NaCl, 5 mM $MgCl_2$) to a final monomeric concentration of 100 nM. The data were collected and processed using the Refeyn AcquireMP and DiscoverMP software.

For Section 2.7 Differential Scanning Fluorimetry (DSF)

The thermal stability of *Lmo*MenD (5 µM) in the absence of ligands (apo) and presence of magnesium and ThDP (300 mM ThDP) was determined using a 96-well plate format (25 µL per well). Using a QuantStudio 3 Real-Time polymerase chain reaction instrument, the change in fluorescence upon thermal denaturation of the protein (25–95 °C) by the SYPRO™ orange dye (Invitrogen; emission maximum 570 nm) was measured in triplicate. Data analysis was performed using the Protein Thermal Shift Software Version 1.4 (ThermoFisher Scientific). The software was used to calculate T_m values by the maximum of first derivative of the thermal denaturation profile curves, which were subsequently plotted in GraphPad Prism 9.

For Section 2.8 UV-Vis activity and DHNA inhibition assays

The UV/Vis-based kinetic assays measuring the consumption of the second MenD substrate, isochorismate (ε_{278} = 8300 M^{-1} cm^{-1}), were adapted from previously established methods (Bashiri *et al.*, 2020, Ho *et al.*, 2025). The isochorismate was enzymatically produced from commercially available chorismate (Sigma Aldrich) using *E. coli* MenF as previously described (Jirgis *et al.*, 2016, Bashiri *et al.*, 2020), and quantified before each use. The reaction buffer contained 50 mM HEPES pH 7.8, 150 mM NaCl and 5mM $MgCl_2$. The standard concentrations of the reaction were 300 nM *Lmo*MenD and 300 µM ThDP with 1 mM 2-oxoglutarate and 25 µM isochorismate added after 2 minutes of incubation at 37 °C to initiate the reaction. These standard concentrations were maintained throughout the kinetic characterisation of *Lmo*MenD, while the concentration of one of the substrates or the cofactor was varied (ThDP: 0-300 µM; oxoglutarate: 0-1 mM; isochorismate: 0-40 µM). Measurements were carried out in triplicate and specific activities (equation 1) were calculated from reaction rates and plotted against the respective ligand concentration. Using GraphPad Prism 9 the Michaelis-Menten (equation 2) model was fitted to the data to determine K_m (constrained: < 0) and v_{max} values, with the latter used to calculate the respective k_{cat} values (equation 3).

$$\text{Specific activity [µmol/mg/min]} = \frac{\text{rate}/(\varepsilon_{278} \cdot l)}{c_{\text{MenD}} \cdot V_{\text{MenD}}} \cdot V_{\text{total}} \qquad (1)$$

$$\text{Specific activity [µmol/mg/min]} = \frac{v_{\max} \cdot c_{\text{ligand}}}{K_m + c_{\text{ligand}}} \qquad (2)$$

$$k_{cat}\ [1/s] = \frac{v_{\max}}{n_{\text{MenD}}} \quad (3)$$

To assess the inhibitory effect of DHNA on MenD activity the percentage change in activity in the presence of 12.5 µM DHNA was determined under the same conditions as described above (at 300 µM ThDP, 1 mM 2-oxoglutarate, and 20 µM isochorismate) based on previously published methods (Stanborough *et al.*, 2023).

Supporting Information: Results and Discussion

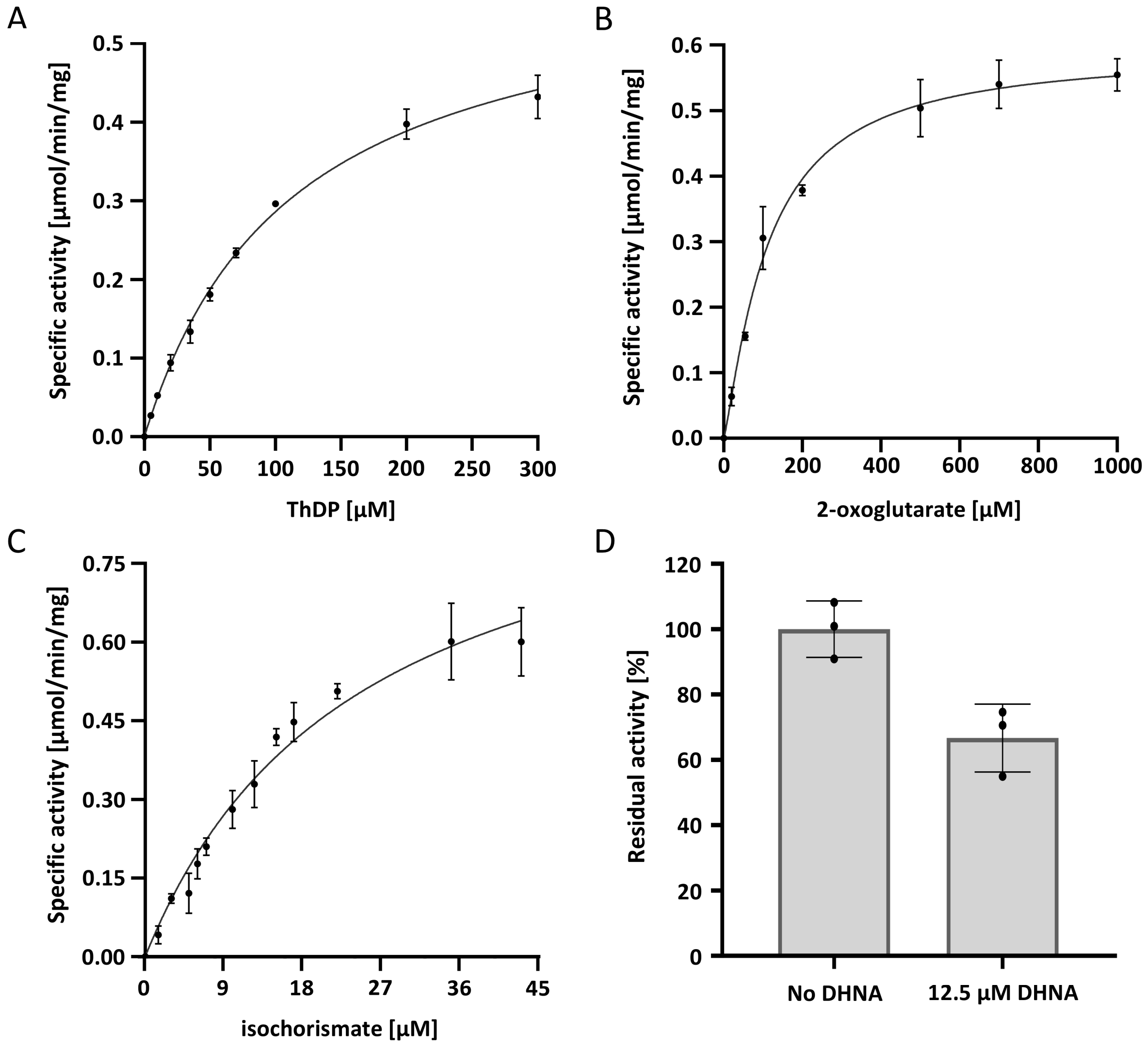

Figure S2 Kinetic characterisation of *Lmo*MenD. The Michaelis-Menten model was fitted to the ThDP (A), oxoglutarate (B) and isochorismate (C) data for the determination of kinetic parameters (Table S1). The addition of 12.5 µM DHNA (D) decreased the activity of *Lmo*MenD by 34% (*Mtb*MenD: 76% (Bashiri *et al.*, 2020); *Sau*MenD: 82% (Stanborough *et al.*, 2023)). In (A), (B) and (C), the data points represent the mean of three technical replicates; the columns in (D) represent the mean of MenD activity normalised to that of the measurement without DHNA, with the three technical replicates shown as data points. In all graphs, the error bars depict standard deviations. All figures were generated using GraphPad Prism 9.

Table S1 Kinetic parameters for LmoMenD derived from Michaelis-Menten kinetics with 95% confidence intervals given in square brackets.

Ligand	K_m [µM]	v_{max} [µmol/min]	k_{cat} [1/s]	R^2
ThDP	111.7 [99.3-126.0]	0.0018 [0.0017-0.0019]	0.65 [0.62-0.69]	0.9929
oxoglutarate	136.2 [110.6-168.0]	0.0016 [0.0015-0.0017]	0.69 [0.65-0.74]	0.9801
isochorismate	22.7 [17.9-29.1]	0.0034 [0.0030-0.0038]	1.05 [0.93-1.20]	0.9652

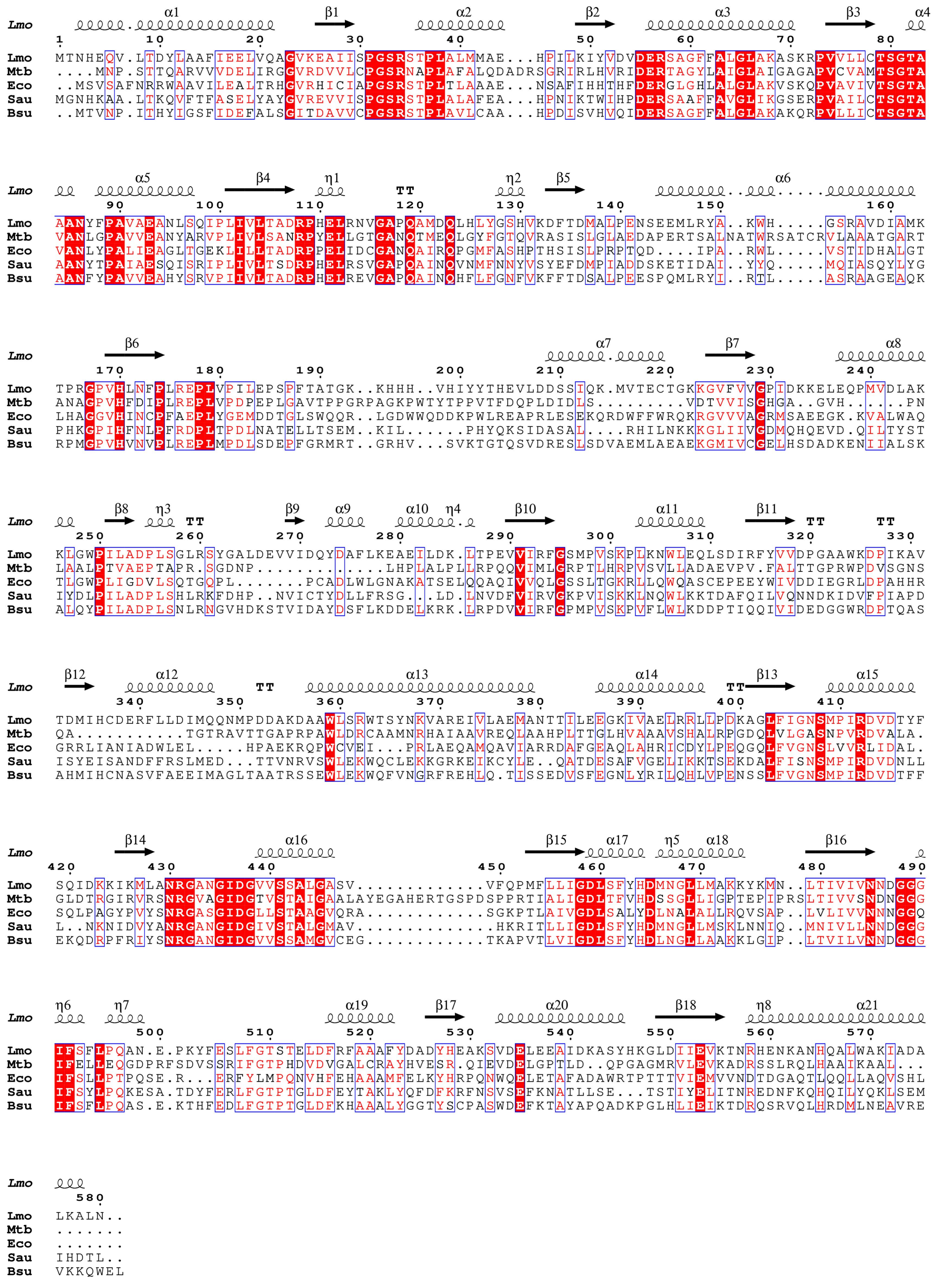

Figure S3 Sequence alignment of structurally characterised MenDs in the PDB generated by Clustal Omega (Madeira et al., 2024) and ESPript 3 (Robert & Gouet, 2014). The sequences of LmoMenD (A0A0H3GD77), MtbMenD (P9WK11), EcoMenD (P17109), SauMenD (Q2FZL7) and BsuMenD (P23970) were obtained from Uniprot, matching all respective PDB entries, and secondary structure elements of our ThDP-bound LmoMenD structure (PDB ID: 9E9B and 9MNN) are depicted. Strictly

conserved (red background, white text) and similar (>0.7 similarity global score; white background, red text) residues across the five MenDs are highlighted.

Table S2 Comparison of structural similarities between LmoMenD (Uniprot ID: A0A0H3GD77) and other structurally characterised MenDs in the PDB.

The sequence identity values were determined via multiple sequence alignment using Clustal Omega (Madeira et al., 2024), whilst the RMSD values across all C_α were derived via PDBeFold (Krissinel & Henrick, 2004).

**Organism**	**Uniprot IDs**	**Sequence identity with *Lmo*MenD [%]**	**PDB ID**	**RMSD across all C_α**
M. tuberculosis	P9WK11	31.61	5ESD	2.00 (chain D)
E. coli	P17109	30.60	2JLC	1.86 (chain A)
S. aureus	Q2FZL7	41.73	7TIN	1.51 (chain A)
B. subtilis	P23970	47. 40	2X7J	1.24 (chain A)

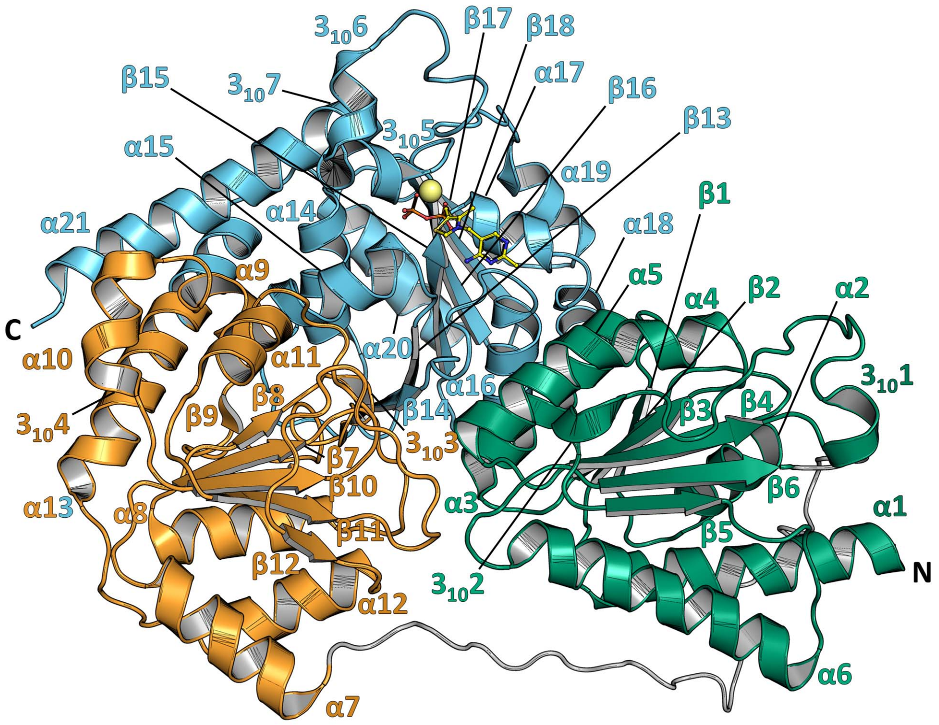

Figure S4 LmoMenD monomer (PDB ID: 9E9B; ThDP and magnesium shown in yellow sticks and sphere) with secondary structure elements of the PYR (green), TH3 (orange) and PP (blue) domains labelled.

Table S3 Comparison of LmoMenD crystals and structures from strain F2365 (apo) and strain 10413a (ThDP- and intermediate I-bound; this study).

Sequence alignment (Figure S6) and identity determination was performed using Clustal Omega (Madeira et al., 2024).

	Apo structure (PDB ID: 3LQ1)	Ligand-bound structures (PDB IDs: 9E9B and 9MNN)
Strain	*L. monocytogenes* strain F2365	*L. monocytogenes* strain 10413a
Uniprot ID	Q71YZ2	A0A0H3GD77
Sequence identity [%]	99.14	
Space group	$P4_322$	$P6_422$
Crystallization condition	2.4 M sodium malonate, pH 7.0, 10% glycerol	9E9B: 12.5% *(w/v)* PEG 1000, 12.5% *(w/v)* PEG 3350, 12.5% MPD, 0.02 M hexane-1,6-diol, 0.02 M butan-1-ol, 0.02 M (*RS*)-propane-1,2-diol, 0.02 M propan-2-ol, 0.02 M butane-1,4-diol, 0.02 M propane-1,3-diol, 0.1 M MOPS/HEPES pH 7.5<br><br>9MNN: 5% *(w/v)* PEG 4000, 20% *(v/v)* glycerol, 0.03 M $MgCl_2$, 0.03 M $CaCl_2$ 0.1 M MES/imidazole, pH 6.3
Resolution [Å]	2.60	2.61 and 2.79
R_{work}/R_{free}	0.222/0.265	0.271/0.324 and 0.303/0.428
Missing regions	A chain: -M1-L1; T189-I200; T381-L384; G488-T512; T556 onwards<br><br>B chain: -M1-L1, A190-I200, G488-T512, T556 onwards	-G2-V1

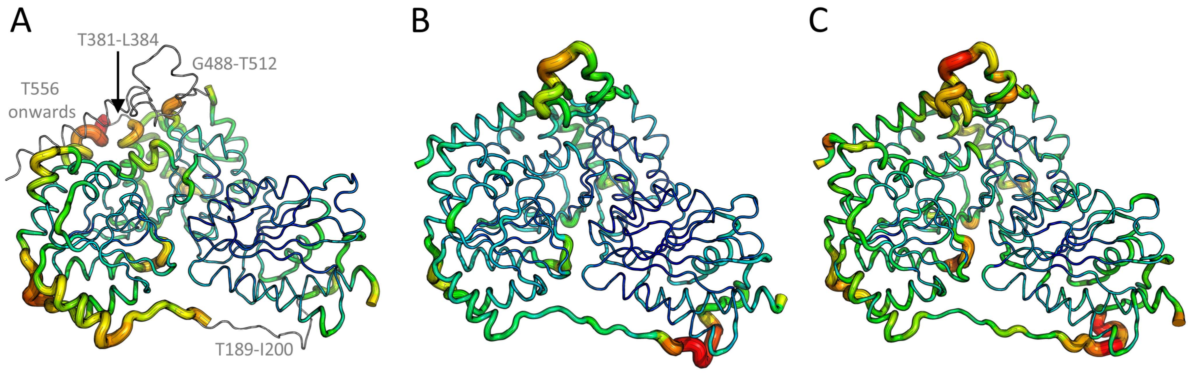

Figure S5 B-factor putty tube images for apo (A; PDB ID: 3LQ1 chain A), ThDP- (B; PDB ID: 9E9B), and intermediate I-bound (C; PDB ID: 9MNN) LmoMenD monomers. To highlight the disordered regions, which are missing in the apo structure (Table S3), the corresponding regions in the ThDP-bound LmoMenD are shown superposed on the apo structure in grey ribbon and labelled.

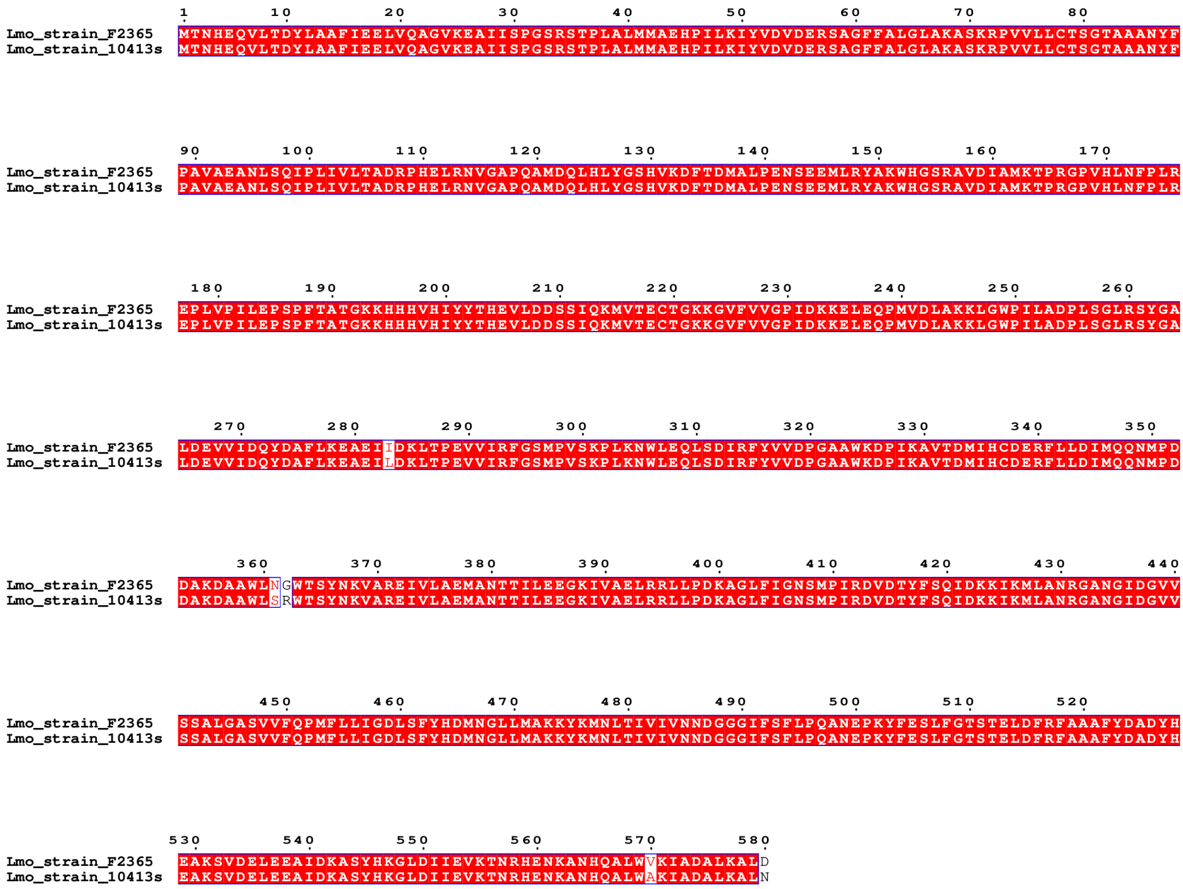

Figure S6 Sequence alignment of *Lmo*MenD from strain F2365 (Uniprot ID: Q71YZ2; PDB ID: 3LQ1) and strain 10413a (Uniprot ID: A0A0H3GD77; PDB IDs: 9E9B and 9MNN) generated by Clustal Omega (Madeira et al., 2024) and ESPript 3 (Robert & Gouet, 2014). Strictly conserved (red

background, white text) and similar (>0.7 similarity global score; white background, red text) residues are highlighted.

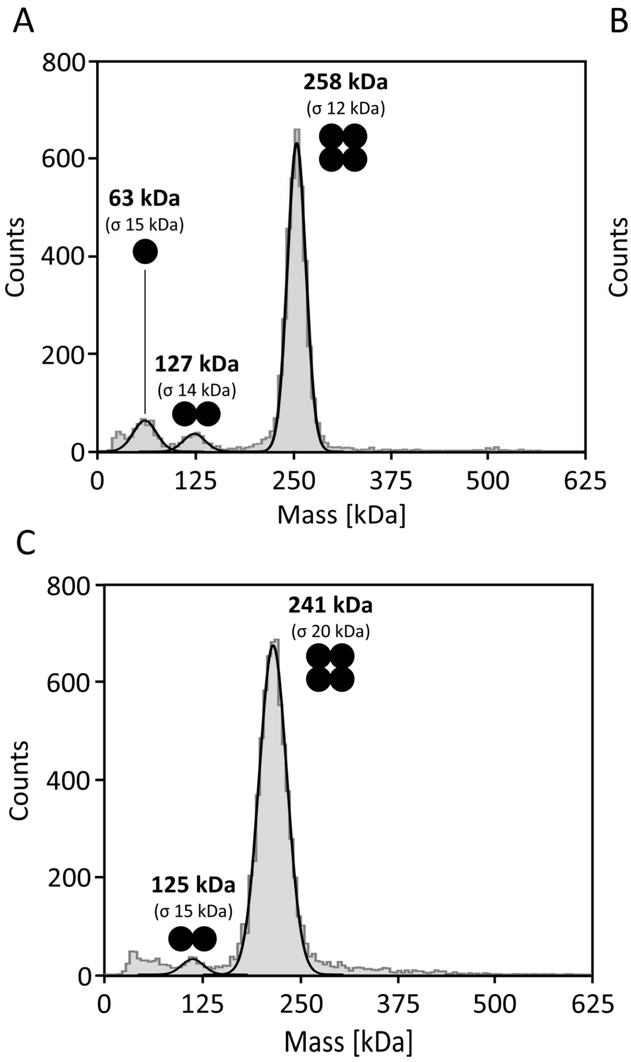

Figure S7 Mass photometry suggested LmoMenD is primarily a tetramer (theoretical monomer: 64.85 kDa; theoretical dimer: 129.7 kDa; theoretical tetramer: 259.4 kDa). Mass photometry histograms in the absence of ligands (A), with 500 µM ThDP (B) and 500 µM ThDP and 200 µM oxoglutarate (C) show that the oligomeric state was unaffected by ligand presence, with LmoMenD predominantly a tetramer across the conditions.

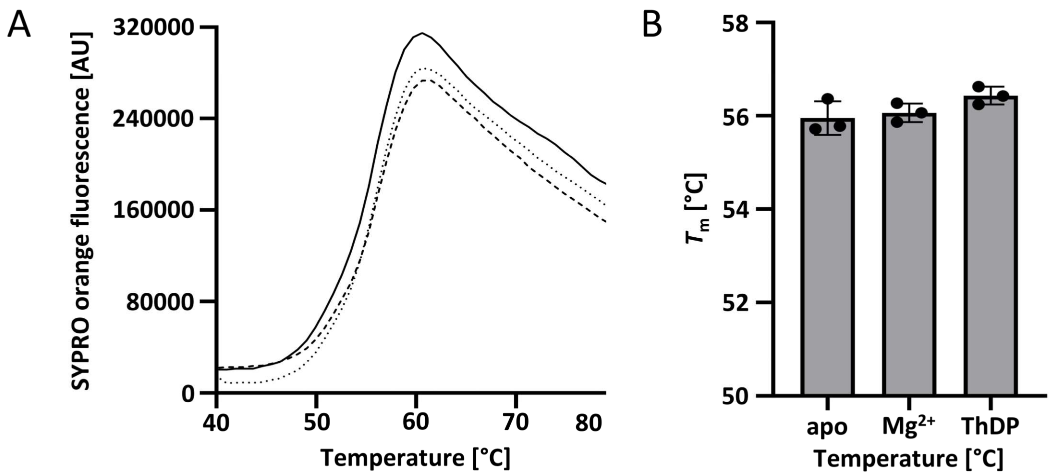

Figure S8 Thermal stability of LmoMenD was independent of ligand presence. Representative thermal denaturation profiles (A) of LmoMenD in the presence and absence of ligands (apo: line; 5 mM Mg^{2+}: dotted; 5 mM Mg^{2+} and 300 mM ThDP: dashed). Comparison of the derived T_m values (B) from triplicate measurements (scatter) show no significant difference in thermal stability, with the error bars depicting the standard deviation.

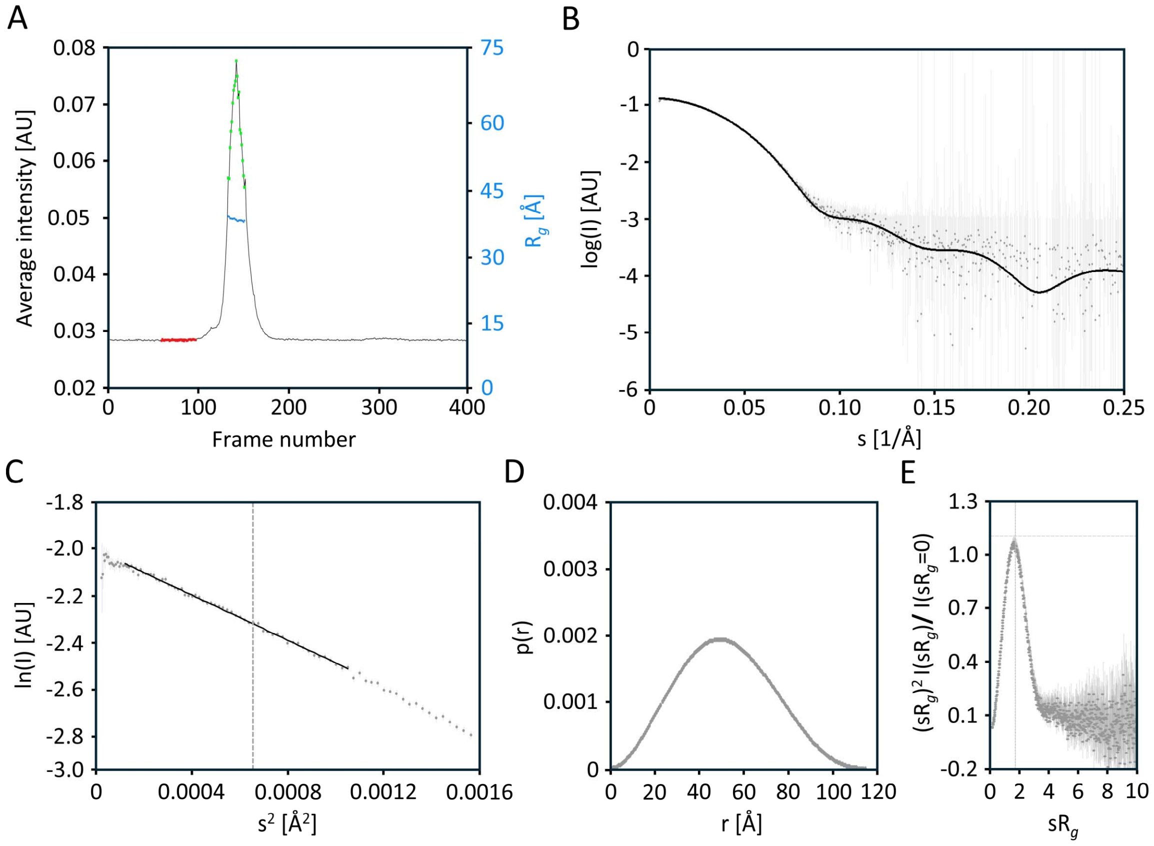

Figure S9 SAXS analysis identified LmoMenD as a tetramer. The CHROMIXS trace (A) shows LmoMenD eluting as one species (as suggested by consistent R_g values; blue) in a single peak from the SEC column (red: buffer frames; green: sample frames). CRYSOL analysis (B) of the LmoMenD scattering profile in solution (grey; R_g: 38.6 Å) identified a close match to the ThDP-bound tetrameric structure (black; PDB ID: 9E9B; χ^2: 0.270; expected R_g (atoms – excluded volume + shell) = 38.3 Å). Together, the Guinier ((C); R_g: 39.0 ± 0.2 Å), distance distribution ((D); R_g/I_0: 39.2 ± 0.2 Å), and dimensionless Kratky ((E); D_{max}: 114 Å) plots suggest LmoMenD to be a globular and folded protein with a consensus molecular weight of 243 kDa calculated via Bayesian inference in PRIMUS (Manalastas-Cantos et al., 2021), matching expectations for a tetrameric LmoMenD (theoretical: 259.4 kDa).

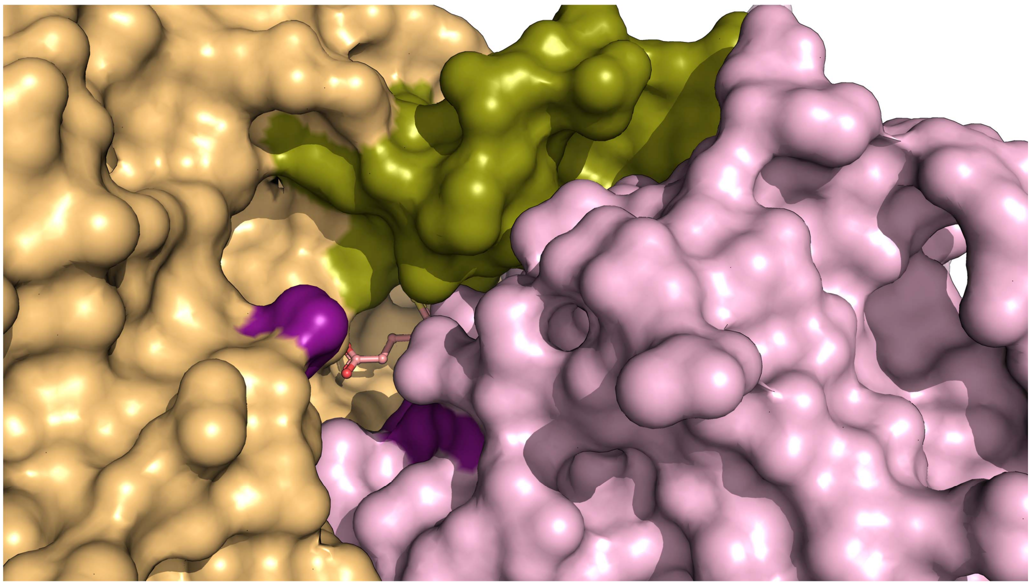

Figure S10 LmoMenD tetramer close-up view on one active site in a dimer (PDB ID: 9MNN). Intermediate I shown in pink sticks within the active site showing the carboxy terminus sticking out into the entranceway. The dimer is shown as a surface rendering (one monomer shown in orange and the other in pink). Key active-site cap residues that close on the active site in the occupied structures that are disordered in apo (residues 488-512) are shown in deep olive, while residues lining that active-site entrance that are inferred by homology to bind to isochorismate/isochorismate portion of intermediate II (S34, R35, R108, Q119, K301) are shown in deep purple.

References

Bashiri, G., Nigon, L. V., Jirgis, E. N., Ho, N. A. T., Stanborough, T., Dawes, S. S., Baker, E. N., Bulloch, E. M. & Johnston, J. M. (2020). *J Biol Chem* **295**, 3759-3770.

Ho, N. A. T., Given, F. M., Stanborough, T., Klein, M., Allison, T. M., Bulloch, E. M., Jiao, W. & Johnston, J. M. (2025). *ChemBioChem* **26**, e202400943.

Jirgis, E. N. M., Bashiri, G., Bulloch, E. M. M., Johnston, J. M. & Baker, E. N. (2016). *Structure* **24**, 1167-1177.

Kirby, N. M., Mudie, S. T., Hawley, A. M., Cookson, D. J., Mertens, H. D., Cowieson, N. & Samardzic-Boban, V. (2013). *Applied Crystallography* **46**, 1670-1680.

Krissinel, E. & Henrick, K. (2004). *Acta Crystallographica Section D: Biological Crystallography* **60**, 2256-2268.

Madeira, F., Madhusoodanan, N., Lee, J., Eusebi, A., Niewielska, A., Tivey, A. R., Lopez, R. & Butcher, S. (2024). *Nucleic acids research* **52**, W521-W525.

Manalastas-Cantos, K., Konarev, P. V., Hajizadeh, N. R., Kikhney, A. G., Petoukhov, M. V., Molodenskiy, D. S., Panjkovich, A., Mertens, H. D., Gruzinov, A. & Borges, C. (2021). *Applied Crystallography* **54**, 343-355.

Robert, X. & Gouet, P. (2014). *Nucleic acids research* **42**, W320-W324.

Ryan, T. M., Trewhella, J., Murphy, J. M., Keown, J. R., Casey, L., Pearce, F. G., Goldstone, D. C., Chen, K., Luo, Z. & Kobe, B. (2018). *Applied Crystallography* **51**, 97-111.

Stanborough, T., Ho, N. A. T., Bulloch, E. M., Bashiri, G., Dawes, S. S., Akazong, E. W., Titterington, J., Allison, T. M., Jiao, W. & Johnston, J. M. (2023). *Philosophical Transactions of the Royal Society B* **378**, 20220035.

Wu, D. & Piszczek, G. (2021). *European Biophysics Journal* **50**, 403-409.
